# PHF-Core Tau as the Potential Initiating Event for Tau Pathology in Alzheimer’s Disease

**DOI:** 10.3389/fncel.2020.00247

**Published:** 2020-09-10

**Authors:** Nabil Itzi Luna-Viramontes, B. Berenice Campa-Córdoba, Miguel Ángel Ontiveros-Torres, Charles R. Harrington, Ignacio Villanueva-Fierro, Parménides Guadarrama-Ortíz, Linda Garcés-Ramírez, Fidel de la Cruz, Mario Hernandes-Alejandro, Sandra Martínez-Robles, Erik González-Ballesteros, Mar Pacheco-Herrero, José Luna-Muñoz

**Affiliations:** ^1^National Dementia BioBank, Departamento de Ciencias Biológicas, Facultad de Estudios Superiores, Universidad Nacional Autónoma de México, Mexico City, Mexico; ^2^Departamento de Fisiología, Escuela Nacional de Ciencias Biológicas, Instituto Politécnico Nacional, Mexico City, Mexico; ^3^School of Engineering and Science, Tecnologico de Monterrey, Toluca, Mexico; ^4^School of Medicine, Medical Sciences and Nutrition, University of Aberdeen, Aberdeen, United Kingdom; ^5^CIIDIR, Instituto Politécnico Nacional, Becario COFAA, Durango, Mexico; ^6^Departamento de Neurocirugía, Centro Especializado en Neurocirugía y Neurociencias México, CENNM, CDMX, Mexico City, Mexico; ^7^Departamento de Bioingeniería, Unidad Profesional Interdisciplinaria de Biotecnología del Instituto Politécnico Nacional (UPIBI-IPN), Mexico City, México; ^8^Neuroscience Research Laboratory, Faculty of Health Sciences, Pontificia Universidad Catolica Madre y Maestra, Santiago de los Caballeros, Dominican Republic

**Keywords:** tau protein, tau pathology, PHF core, truncation, phosphorylation, conformational changes, paired helical filament, neurofibrillary tangles

## Abstract

Worldwide, around 50 million people have dementia. Alzheimer’s disease (AD) is the most common type of dementia and one of the major causes of disability and dependency among the elderly worldwide. Clinically, AD is characterized by impaired memory accompanied by other deficiencies in the cognitive domain. Neuritic plaques (NPs) and neurofibrillary tangles (NFTs) are histopathological lesions that define brains with AD. NFTs consist of abundant intracellular paired helical filaments (PHFs) whose main constituent is tau protein. Tau undergoes posttranslational changes including hyperphosphorylation and truncation, both of which favor conformational changes in the protein. The sequential pathological processing of tau is illustrated with the following specific markers: pT231, TG3, AT8, AT100, and Alz50. Two proteolysis sites for tau have been described—truncation at glutamate 391 and at aspartate 421—and which can be demonstrated by reactivity with the antibodies 423 and TauC-3, respectively. In this review, we describe the molecular changes in tau protein as pre-NFTs progress to extracellular NFTs and during which the formation of a minimal nucleus of the filament, as the PHF core, occurs. We also analyzed the PHF core as the initiator of PHFs and tau phosphorylation as a protective neuronal mechanism against the assembly of the PHF core.

## Introduction

**FIGURE 1:**
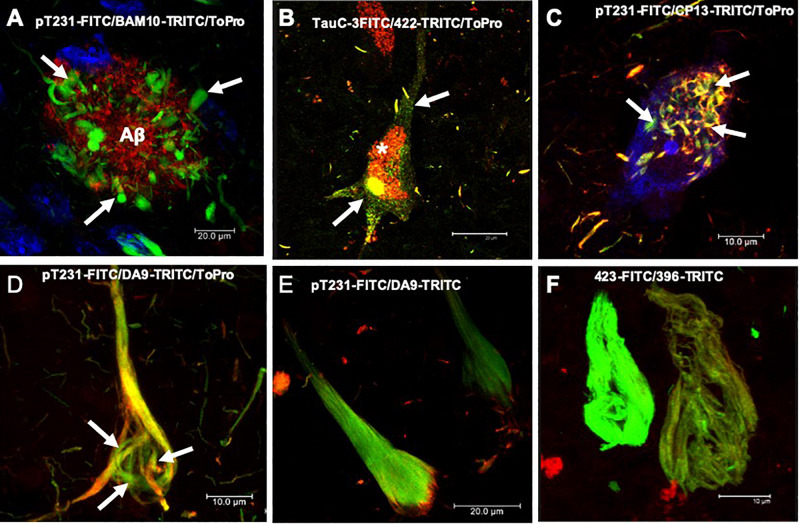
Histopathological lesions in Alzheimer’s disease (AD) brain. **(A)** Amyloid plaque stained using the BAM10 antibody (*red channel*), with associated dystrophic neurites (*arrows*), and nuclei stained with To-Pro (*blue*). **(B–F)** Evolution of the aggregation of the tau protein recognized by different antibodies directed against phosphorylated and truncated tau proteins. (B) Pre-neurofibrillary tangle (NFT) characterized by a diffuse granular staining in the neuronal soma (TauC-3, *green channel*; 423, *red channel*, *arrows*). Perinuclear immunoreactivity is observed. Lipofuscin is autofluorescent in the red channel (*asterisk*). **(C)** Small tangles (bead-like structures, *arrows*) visualized using two antibodies directed against the phosphorylated tau protein (pT231, *green channel*; CP13, *red channel*). **(D,E)** NFTs at different stages of aggregation. **(D)** NFT with a fibrillar structure in the form of a trabecula around the nucleus (*arrows*). **(E)** Compact NFT, where the PHFs have invaded the entire soma and displaced the nucleus from its original position, and visualised using pT231, which recognises a phospho-epitope within the mid domain (DA9). **(F)** Extracellular NFTs, the last stage of aggregation of the tau protein. In the neuronal soma, this structure is much looser, lacking a cell membrane and a nucleus. It consists of the minimum core of the filament (PHF core) and reacts with antibody 423, which recognizes truncation at Glu-391 (*green channel*) and phospho-tau S396 (*red channel*). Images obtained with a Leica SP8 confocal microscope.

**FIGURE 2:**
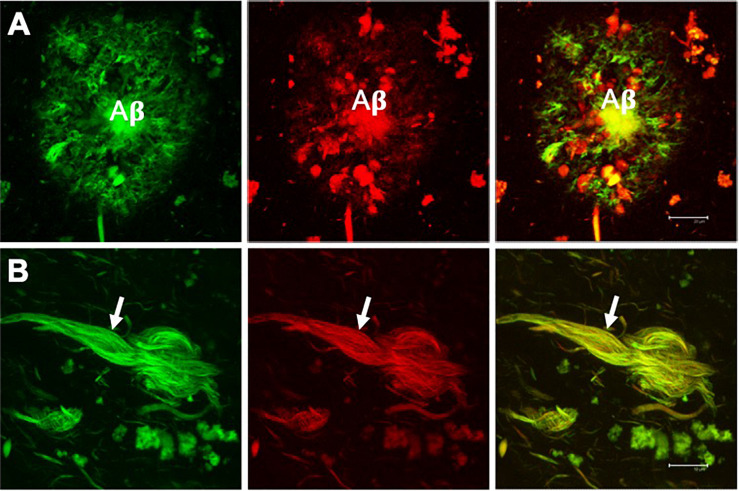
Double fluorescent staining of a case with Alzheimer’s disease. Amyloid plaque **(A)** and neurofibrillary tangle (NFT) **(B)** evidenced by the dye thioflavin S (TS, *green channel*) and thiazine red (TR, *red channel*). Both markers co-locate, giving a yellow color in the merged channel. TS and TR demonstrate the fibrillar state with the β-folded conformation of the amyloid β-peptide (Aβ) and the tau protein assembled into filaments. Scale bar **(A)** 20 μm, **(B)** 10 μm. Images obtained with a Leica SP8 confocal microscope.

**FIGURE 3:**
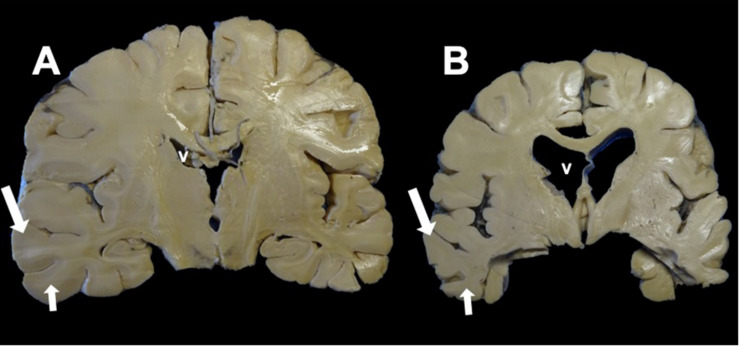
Coronal section of the brain. **(A)** Control. **(B)** Alzheimer’s disease (AD). Macroscopic morphological changes are observed. In AD, there is a reduction in the size of the brain associated with neuronal death caused by neurofibrillary tangles (NFTs). The ventricles (*v*) and the grooves between the convolutions widen, and there is a considerable reduction in the thickness of the gray matter (*large arrow*) and white matter (*small arrow*).

The elderly population is increasing globally, and this leads to the increased prevalence of neurodegenerative diseases typical of this age group. Moderate and severe Alzheimer’s disease (AD) can be clinically diagnosed with a high degree of certainty. However, at a preclinical or early stage, symptoms shared with other neurodegenerative diseases make the diagnosis of AD difficult. 1Clinically, AD is characterized by progressive memory loss and impaired cognitive functions (judgment, behavior, and language). Neuritic plaques (NPs) (Figure 1A) and neurofibrillary tangles (NFTs) (Figures 1B–F), which can be stained by thiazin red (TR) and thioflavin S (TS) (Kelenyi, 1967; Stiller et al., 1970; Mena et al., 1995; Luna-Munoz et al., 2008; Figure 2), are histopathological lesions in brains with AD (Perl, 2010). At a macroscopic level, decreases in brain size (Figures 3A,B), gray matter (Figure 3B, arrows), and white matter (Figure 3B, small arrows) are observed. A substantial increase in ventricular volume and significant atrophy in the brain convolutions and the hippocampus are also observed (Figure 3B). NFTs are associated with neuronal death in AD. They consist of abundant intracellular paired helical filaments (PHFs) whose main constituent is tau protein. To aggregate into PHFs, tau dissociates from microtubules, an event favored by specific modifications (Crowther and Wischik, 1985; Wischik and Crowther, 1986; Wischik et al., 1992). In this review, we discuss the posttranslational mechanisms of the phosphorylation and truncation of tau protein that are associated with the formation of PHFs. We conclude from our review that pharmacological therapy for AD should not be directed against phosphorylated tau.

## Neuritic Plaques

**FIGURE 4:**
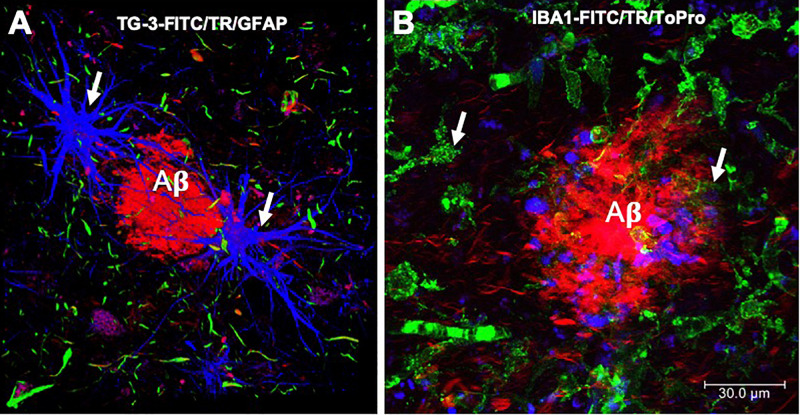
Immunofluorescence of amyloid plaques. **(A)** Amyloid β-peptide (Aβ) deposit recognized by thiazine red (TR, *red color*), which is bordered by glial cells (GFAP, *blue color*) and by dystrophic neurites, recognized by the antibody that reacts with phosphorylated tau protein (TG-3, *green color*). **(B)** Amyloid plaque is recognized by the TR dye (*red color*). At the periphery, microglial cells are recognized by the Iba-1 antibody (*green color*) and the cell nuclei with To-Pro (*blue color*). Images obtained with a Leica SP8 confocal microscope.

Neuritic plaques are made up of soluble or insoluble (Figure 1A) extracellular deposits of amyloid β-peptide (Aβ). NPs are bordered by filiform structures that are the dystrophic neurites, which are part of the dendrites and axons of neurons (Guevara et al., 1998; Espinosa et al., 2001) and glial (Figure 4A; DaRocha-Souto et al., 2011; Serrano-Pozo et al., 2011) and microglial cells (Figure 4B; Hayes et al., 2002; Jekabsone et al., 2006; Lee and Landreth, 2010). Aβ is formed from the proteolytic processing of a transmembrane protein called the amyloid precursor protein (APP) (Kapaki et al., 2005). APP can be processed in two ways: one physiological or non-amyloidogenic and the other pathological or amyloidogenic. In the non-amyloidogenic pathway, APP is cleaved by α-secretase in its N-terminal ectodomain (sAPPα), leaving the C-terminal α-CTF fragment anchored in the membrane. Subsequently, α-CTF is cut by the action of γ-secretase, giving rise to fragments p3 and AICD (APP intracellular domain). In the amyloidogenic pathway, β-secretase initiates APP proteolysis by cutting the ectodomain called sAPPβ (soluble peptide APPβ). The membrane-anchored fragment or β-CTF is subsequently cut by γ-secretase, generating Aβ. While Aβ peptides 1-40 and 1-42 are the main constituents of the NPs, Aβ1-42 is the first to be deposited and has greater ease of adding and polymerizing under physiological conditions (Jarrett et al., 1993; Iwatsubo et al., 1994). Aβ undergoes post-translational modifications such as oxidation, phosphorylation, glycosylation, pyroglutamination, isomerization, or racemization. These modifications may favor Aβ polymerization, toxicity, and inflammatory activity observed in cases of AD (Polanco et al., 2018).

## Neurofibrillary Tangles

The presence of NFTs in the hippocampus follows a stereotyped pattern described in six stages (Braak and Braak, 1991). In stages I and II, NFTs are observed in the transentorhinal cortex and the adjacent area of the entorhinal cortex II. They are considered the preclinical stages in the absence of clinical symptoms. In stages III and IV, NFTs have invaded mostly the entorhinal cortex II, subiculum, and CA1. At these stages, the first clinical symptoms, or memory loss, begin. Stages V and VI are characterized by a complete invasion of the hippocampus, layer IV of the entorhinal cortex, and the neocortex. These latter stages correspond to an advanced phase of AD. This pathological progression is important in the postmortem diagnosis of AD and characterization of the stages in its development.

## Tau Protein

**FIGURE 5:**
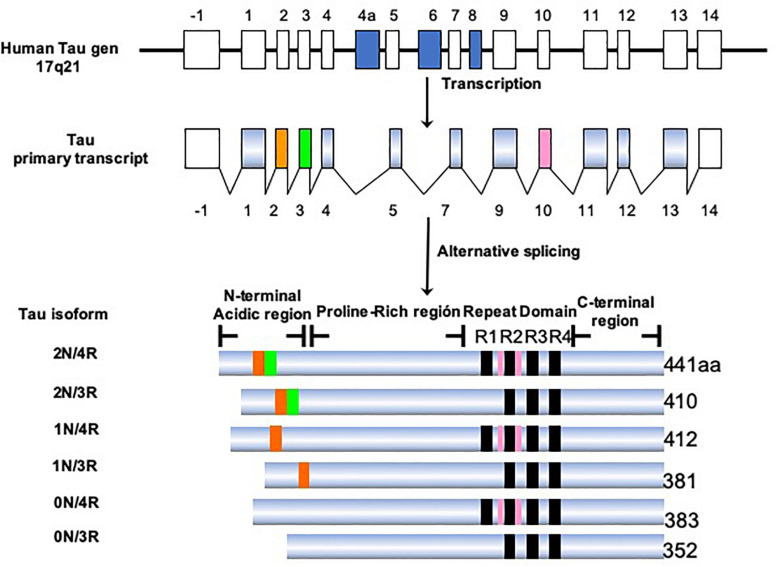
Isoforms of the tau protein. The tau protein gene is located on the long arm of chromosome 17, which generates six isoforms by alternative processing. The longest isoform in the CNS is 441 amino acids and the shortest is 352 amino acids. The length depends on the presence/absence of inserts in the amino terminal portion and the presence of three or four repeated domains.

Tau protein belongs to the family of microtubule-associated proteins (MAPs) (Goedert et al., 1988; Lee et al., 1988; Himmler, 1989). In humans, tau is encoded by a single-copy gene located on chromosome 17q21.3 (Neve et al., 1986). This gene has 16 exons, of which exons 2, 3, 4A, 6, 8, 10, and 14 can be alternatively spliced (Figure 5). This processing generates six isoforms of tau in the central nervous system (CNS), ranging from 352 to 441 amino acids in length (Goedert et al., 1989b). Structurally, the tau molecule is highly elastic, without secondary structure (Schweers et al., 1994). It presents two domains: an amino terminal domain named “projection domain,” composed of an acidic region and a proline-rich region. The carboxy-terminal domain consists of the “microtubule-binding domain,” which contains three (3R) or four tandem repeats (4R) of 31 or 32 amino acids and a C-terminal tail. The additional repeat region is encoded by exon 10. It is the repeat domain of tau that is vital for its ability to polymerize into filaments, and it is highly sensitive to phosphorylation (Steiner et al., 1990). The tau isoforms differ in the presence of N-terminal inserts (0, 1, or 2) and the number of C-terminal repeats (3R or 4R). During the fetal and early developmental stages, 3R tau isoforms are predominant, whereas both the 3R and 4R isoforms can be found in adult brains (Goedert et al., 1989a; Andreadis et al., 1992). The 4R isoform is about 40-fold more efficient at binding microtubules than the 3R isoform. Thus, the 3R tau would allow greater cytoskeletal plasticity in the growing immature neurons of the fetal brain (Lindwall and Cole, 1984; Bramblett et al., 1993). It has been shown that the 4R/3R ratio in normal and AD brains are 1:1 and approximately 2:1, respectively (Goedert et al., 1989a). Alternative splicing of exon 10, which impacts on the expression of the 3R and 4R isoforms, could be related to the pathogenesis of tauopathies (Liu and Gong, 2008; Zhou et al., 2008). It has been demonstrated that the presence of these isoforms differs according to the type of tau deposit and corresponds to the morphological structure of NFTs (Ginsberg et al., 2006). For example, AD is characterized by 95% PHFs and 5% straight filaments (SFs), whereas in Pick’s disease the filaments are predominantly SFs. Tau-positive neurons, which exhibit diffuse cytoplasmic tau without apparent fibrillary structures (pre-tangle neurons), appear to be 3R-negative/4R-positive. Intracellular NFTs with typical fibrillary structures contain equal amounts of 3R and 4R isoforms. Structures that are 3R-positive/4R-negative would correspond to extracellular ghost tangles. 3R tau-positive lesions are abundant in the areas in which tau deposition begins early and increase with disease progression (Ginsberg et al., 2006). In contrast, 4R tau-positive lesions appear in the regions in which tau deposition starts later. In this sense, an orchestrated regulation would change the tau isoform as the AD progresses (Jakes et al., 1991; Uchihara et al., 2012; Uchihara, 2014; Uematsu et al., 2018). It remains to be clarified how the synthesis of the different isoforms is regulated and exactly how the 3R and 4R tau isoforms affect the progression of AD.

## Posttranslational Modifications Involved in the Genesis of PHFs

Tau protein undergoes a number of posttranslational modifications: phosphorylation, truncation, acetylation, methylation, glycosylation, nitration, glycation, and SUMOylation. The hyperphosphorylation and truncation of tau have been extensively studied in relation to the genesis of PHFs.

### Tau Hyperphosphorylation

**FIGURE 6:**
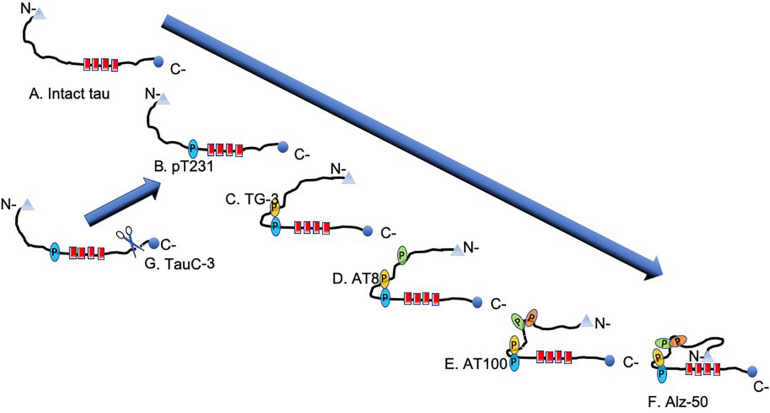
Scheme of the stages of molecular tau processing. **(A)** Intact tau molecule shown. **(B)** On intact tau protein occurs the first phosphorylation in Thr231. This phosphorylation is decisive for the first conformational change evidenced by the TG-3 antibody **(C)**. **(D)** The amino acids 202 and 205 (AT8) are phosphorylated, which favors the phosphorylation in amino acids 212 and 214 (AT100). It generates the second pathological conformational change in tau **(E)**. The set of these two regional conformational changes favors the folding of the N-portion, causing the regional conformational change recognized by the Alz-50 antibody (epitopes 2–10 and 312–322) **(F)**. **(G)** In pre-neurofibrillary tangles (NFTs), there is a high activity of caspase-3, which acts once the tau protein is phosphorylated in pT231.

The concentration of phosphorylated tau is increased by two to threefold in AD compared with healthy controls (Blennow et al., 1995; Vigo-Pelfrey et al., 1995). Tau protein has 85 feasible phosphorylation sites: 45 serine, 35 threonine, and five tyrosine residues. Of these, 30 sites appear to be abnormally phosphorylated (Figure 6). Phosphorylation causes tau to lose affinity for β-tubulin, microtubule depolymerization, and pathological aggregation (Noble et al., 2013; Ercan-Herbst et al., 2019). Phosphorylation of the serine residues 235, 262, 293, 324, and 356 favors detachment of tau from tubulin (Drewes et al., 1995; Liu et al., 2007). The main kinases involved in this process are the glycogen synthase kinase 3 beta (GSK-3β), cell division protein kinase 5 (CDK5), AMP-activated protein kinase (AMPK), protein kinase A (Guillemin et al., 2005), and FYN (Mandelkow et al., 1992; Morishima-Kawashima and Kosik, 1996; Andorfer and Davies, 2000; Lee et al., 2004; Thornton et al., 2011; Mairet-Coello et al., 2013). The participation of these enzymes in neurodegeneration, however, remains to be established (Noble et al., 2013).

### Tau Truncation

Tau can undergo proteolysis by various enzymes *in vitro*: caspase-6, which cleaves tau between amino acids 13–14 and 402–403; caspase-3 (25–26 and 421–422); calpain (44–45, 230–231, and 242–243); ADAM10 (152–153); thrombin (155–156); and chymotrypsin (197–198) (Amadoro et al., 2020). For the identification of the truncation of tau at aspartate 421, Gamblin et al. (2003a) developed the monoclonal antibody TauC-3. Its characterization on AD brain tissue showed it to have a high affinity for NFTs and dystrophic neurites (Guillozet-Bongaarts et al., 2005). Meanwhile, Rissman et al. (2004) recognized the same truncation using a polyclonal antibody, which showed reactivity in neurons lacking developed fibrillar structures. In contrast, the minimal nucleus, or PHF core, of the protease-resistant filaments (Wischik et al., 1985) is recognized by the monoclonal antibody 423. This PHF core consists of a fragment of the tau protein with 92–95 amino acids, ending at glutamate 391 (Glu-391). It is made up of three and a half tau domains, phase-shifted with respect to the tandem repeat domains, and is characterized by truncation at Glu-391 and, being highly insoluble, highly resistant to degradation by either formic acid or pronase (Novak et al., 1989; Wischik et al., 1992). Isolation and subsequent characterization of the PHF core showed it to have a C-shaped sub-domain repeating within a helical structure (Wischik et al., 1985, 1988a,b, 1992; Wischik and Crowther, 1986; Mena et al., 1996). Recently, the molecular structure of these C-shaped sub-domains of the PHF core has been established by cryo-electron microscopy (Fitzpatrick et al., 2017). It has been observed *in vitro* that the PHF-core tau is able to form PHFs (Al-Hilaly et al., 2017) and that its overexpression is capable of inducing cell death by apoptosis in COS cell cultures (Fasulo et al., 1998). In AD brains, the PHF core co-locates with intact tau and phosphorylated tau, from pre-NFTs to extracellular NFTs (Flores-Rodriguez et al., 2015). This structure is found in the different stages of the pathological processing of tau (intact, phosphorylated, and tau with conformational changes). It has been suggested that the PHF core favors the capture of intact phosphorylated tau molecules, preventing the neuron from perceiving the truncation in Glu-391 and its apoptosis (Fasulo et al., 1998, 2005).

## Conformational Changes of the Tau Protein

### Regional Conformational Changes

Posttranslational pathological processing of the tau protein includes regional conformational changes dependent on phosphorylation (Jicha et al., 1997b; Zheng-Fischhofer et al., 1998) and structural changes (Carmel et al., 1996; Jicha et al., 1997a, 1999) dependent on these regional conformational changes (Luna-Munoz et al., 2005, 2007). Some regional conformational changes are characterized by phosphorylated amino acid residues. One of these conformational changes results from phosphorylation at position threonine 231 (Thr-231) and serine 235 (Ser-235), recognized by the TG-3 antibody (Wolozin et al., 1986; Figure 6). Meanwhile, the AT100 antibody also recognizes a regional conformational change dependent on the phosphorylation of amino acid residues 202 and 205 and, additionally, 212 and 214. Previous studies, using recombinant tau, determined that the phosphorylation of residues 202 and 205 (recognized by the AT8 antibody) occurs before the phosphorylation of amino acids 212 and 214. In contrast, if amino acids 212 and 214 are phosphorylated first, no regional conformational change occurs, and AT100 shows no reactivity (Zheng-Fischhofer et al., 1998). It has been observed that the epitopes recognized by the TG-3 (phospho-tau 231–235) antibody are very stable during the evolution of the NFT, similar to pT396 (phospho-tau at amino acid 396), because they are closer to the contiguous portion of the PHF core.

### Structural Conformational Changes

There is a structural conformational change of tau that depends on an intact amino terminal (amino acids 2–10), and the third repeat (312–342). This conformation can be seen using Alz50, an antibody that has been associated with the initial stage of the pathological processing of tau (Jicha et al., 1997a).

## Sequence of Molecular Events During the Aggregation of Tau Protein in PHF Formation

**FIGURE 7:**
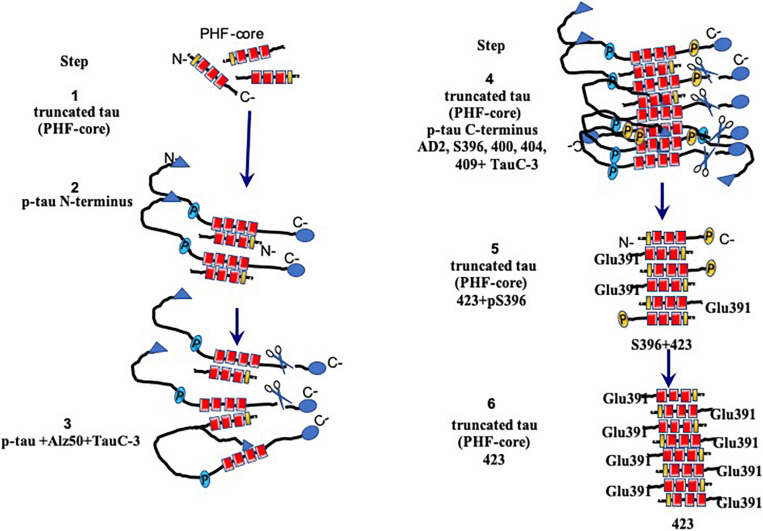
Model for the assembly and processing of tau proteins in paired helical filaments (PHFs). Neurons that have not yet been affected express the intact tau protein associated mainly with microtubules in axons. Braak stages I–IV are the early stages prior to fibrillary inclusions. Stages I and II represent the presence of a PHF core (circa 95-amino acid residue fragment). Stage III is characterized by the cytoplasmic aggregation of the tau molecules, favored by the nuclear fragments of tau. PHFs begin their formation with intact and tau molecules phosphorylated in the N-terminus. This stage corresponds to the presence of the granular tau protein in the neuronal cytoplasm seen by confocal microscopy. The PHF core is masked by intact and phosphorylated tau molecules. In stage IV, the truncation between the amino acids Asp-421/Ser-422 appears. This truncation is observed from the granular stages. In stage V, intracellular PHFs and fibrils are initiated, phosphorylated in both their N- and C-terminal portions, which are recognized with antibody 423 (Glu-391 truncation). The fibrillar nature is confirmed by the affinity of the thiazine red (TR) dye in stage VI. The N-terminal portions are removed. As the neurofibrillary tangle (NFT) becomes extracellular, the structures are highly insoluble and are exclusively immunoreactive with antibody 423, with only occasional epitopes of pS396 available.

Pathological aggregation of the tau protein follows a series of structural steps ranging from the formation of a pre-NFT to the formation of an extracellular NFT (Figures 1B–F, 7). The pre-NFT, characterized by a diffuse granular form in the cytoplasm (Figure 1B), results in a perinuclear staining recognized by some phosphorylated epitopes in the amino terminus and by an intact tau protein. This aggregation has no affinity for the TR dye as it is an assembly marker. The next stage is characterized by the presence of small dense aggregates of tau or small tangles (Figure 1C), which are related to the TR dye. These small packages converge and form a trabecula in the neuronal soma (Figure 1D). This structure fills the neuronal body, forming an intracellular NFT, which displaces the cell nucleus from its original central position toward the periphery (Figure 1E). Finally, these filaments are exposed as extracellular NFTs, which is only detected using the TR dye and the antibody 423 (truncation in Glu-391). This stage is characterized by a loose fibrillar structure in which the cell membrane and the nucleus have been lost (Figure 1F; Mena et al., 1991; Galvan et al., 2001; Luna-Munoz et al., 2007). At a molecular level, late events can be seen in NFTs, such as a structural conformational change or truncation in Asp-421 and Glu-391. Truncation at Asp-421 occurs after the conformational change recognized by the antibody Alz-50 (when the tau protein is perfectly assembled in the filament) and culminates with the presence of truncation at Glu-391 (Garcia-Sierra et al., 2003; Guillozet-Bongaarts et al., 2005; Basurto-Islas et al., 2008). However, this model does not take into account of phosphorylation state. The molecular events associated with tau phosphorylation follow a well-defined order (Figure 6). However, it is difficult to follow the phosphorylation of tau in the NFTs since, in these structures, the tau protein is found simultaneously in different states of expression and aggregation. Thus, the earliest events are studied in the pre-NFTs (Figure 6A). It has been suggested that the first step is the phosphorylation of the tau protein at Thr-231 (Figure 6B), followed by the phosphorylation of Ser-235, which leads to the first regional conformational change, detectable using the TG-3 antibody (Figure 6C). Subsequently, amino acid residues 202 and 205 become phosphorylated (epitopes recognized by the AT8 antibody) (Figure 6D). This modification involves a second phase of phosphorylation at amino acids 212–214 and a second regional conformational change identified by the AT100 antibody (Figure 6E). These changes lead to a structural conformational change recognized by the Alz50 antibody (Figure 6F; Luna-Munoz et al., 2007), which requires an intact N-terminus (Carmel et al., 1996; Jicha et al., 1999). The truncation at Asp-421 is observed immediately after phosphorylation at Thr-231 (Luna-Munoz et al., 2007; Figure 6G). This suggests that the presence of the truncated tau protein at Asp-421 favors filament polymerization (Gamblin et al., 2003a,b; Yin and Kuret, 2006).

## Template Tau Protein in PHF

We have been investigating the relationship between the different species of phosphorylated and truncated tau protein and the mechanism whereby tau assembles into insoluble and stable PHFs over a period of several years. We have been analyzing pre-tangle cells (Figure 1B, arrow), in which the first steps of non-fibrillary aggregation of the tau protein arise in AD. On the basis of our morphomolecular analysis, we propose the following steps in PHF assembly (Figure 7).

(1) The presence of a PHF core (297–391) is a highly toxic truncated tau species.
(2) A specific cascade of phosphorylation on the N-terminus of the tau protein (Figure 6).
(3) Truncation of the C-terminus by caspase-3.
(4) Aggregation and oligomerization of all species of tau.
(5) Assembly of tau protein in PHFs.

The first event that occurs in the formation of PHFs, and with it the NFTs, would be represented by the appearance (*via* an unknown origin) of subunits of the PHF core (Figure 7, step 1). The toxicity of the truncated tau (92–95 amino acids) is associated with the high affinity of the intact tau and the phosphorylated tau to this small fragment (Figure 7, step 2), which would trigger an immediate neuroprotective mechanism. This would be reflected by the hyperphosphorylation of the tau molecule in a failed attempt to hide the PHF core and avoid the kidnapping of the molecules of intact tau. Unfortunately, in AD, the protective mechanism that might be involved in phosphorylated tau protein would only favor that there are more molecules available for its sequestration and the formation of PHF, which represents, finally, a polymer made up of fragments of tau in an intracellular NFT (Figure 7, steps 3 and 4). In extracellular NFTs, all phosphorylation is lost as a result of proteolysis, during which the PHF core becomes exposed (Figure 7, steps 5 and 6). There are findings suggesting that phosphorylation of the tau protein may have a protective role and be non-toxic (Castellani et al., 2008; Congdon and Duff, 2008; Luna-Munoz et al., 2013; Flores-Rodriguez et al., 2015). This implies that NFTs serve as a protective structure.

## Future Study of Tau Protein

The complete functions of the tau protein remain to be elucidated. Tau is a stabilizing microtubule-associated protein. In the nucleus, this protein protects the DNA in situations of cellular stress; in the nucleolus, it favors the nucleolar function (Sjoberg et al., 2006), the process of mitosis (Flores-Rodriguez et al., 2019) and meiosis (Inoue et al., 2014). Tau has been previously observed in non-neuronal organs such as the heart, skeletal muscle, lung, or skin and in different states of non-pathological phosphorylation (Gu et al., 1996; Zhou et al., 2020). This suggests that tau phosphorylation may be involved in functions yet to be established. Therapeutic approaches to prevent tau phosphorylation could cause problems in these organs. Truncation at Glu-391 appears to be a good differential marker between AD and other neurodegenerative pathologies.

## Conclusion

The aggregation and polymerization of the tau protein has been suggested to be a response to pathological events that occur early in neurons and the brain. Tau phosphorylation and NFT formation seem to act as a protective event against the minimal nucleus of the filament (PHF core), which functions as a prion and is highly toxic. Taking into account that NFTs are closely correlated with the cognitive deterioration of patients, it is vitally important to look for other proteins that can define the early onset of AD. Whereas the development of drugs directed against the phosphorylation of the tau protein may involve certain risks, targeting the aggregation of tau proteins with compounds that fail to affect the normal association of tau with microtubules offers another therapeutic target (Wischik et al., 2014; Wilcock et al., 2018). Continued donation of tissue for research will be of utmost importance since it will allow a better understanding of these pathological events and, ultimately, bring hope of discovering effective methods to both diagnose and cure AD.

## Data Availability Statement

All datasets presented in this study are included in the article/supplementary material.

## Ethics Statement

The studies involving human participants were reviewed and approved by Ethics Committee, Facultad de Estudios Superiores UNAM. Written informed consent was obtained for the brain donation to National Dementia BioBank.

## Author Contributions

MP-H and JL-M contributed to the idea formulation, writing, and revision of the manuscript. CH contributed to the writing and revision of the manuscript. NL-V and BC-C contributed to the images and revision of the manuscript. MO-T, IV-F, PG-O, LG-R, FC, SM-R, and EG-B contributed to the revision of the manuscript. All authors contributed to the article and approved the submitted version.

## Conflict of Interest

CH is an officer of TauRx Therapeutics Ltd. and an inventor on patents relating to tau-aggregation inhibitors. The remaining authors declare that the research was conducted in the absence of any commercial or financial relationships that could construed as a potential conflict of interest.
